# Effects of matrix metalloproteinases on the fate of mesenchymal stem cells

**DOI:** 10.1186/s13287-016-0393-1

**Published:** 2016-09-09

**Authors:** Sami G. Almalki, Devendra K. Agrawal

**Affiliations:** Department of Clinical and Translational Science, Creighton University School of Medicine, CRISS II, Room 510, 2500 California Plaza, Omaha, NE 68178 USA

**Keywords:** Mesenchymal stem cells, Matrix metalloproteinases, Extracellular matrix, Osteogenic differentiation, Adipogenic differentiation, Chondrogenic differentiation, Migration, Angiogenesis, Proliferation

## Abstract

Mesenchymal stem cells (MSCs) have great potential as a source of cells for cell-based therapy because of their ability for self-renewal and differentiation into functional cells. Moreover, matrix metalloproteinases (MMPs) have a critical role in the differentiation of MSCs into different lineages. MSCs also interact with exogenous MMPs at their surface, and regulate the pericellular localization of MMP activities. The fate of MSCs is regulated by specific MMPs associated with a key cell lineage. Recent reports suggest the integration of MMPs in the differentiation, angiogenesis, proliferation, and migration of MSCs. These interactions are not fully understood and warrant further investigation, especially for their application as therapeutic tools to treat different diseases. Therefore, overexpression of a single MMP or tissue-specific inhibitor of metalloproteinase in MSCs may promote transdifferentiation into a specific cell lineage, which can be used for the treatment of some diseases. In this review, we critically discuss the identification of various MMPs and the signaling pathways that affect the differentiation, migration, angiogenesis, and proliferation of MSCs.

## Background

The extracellular matrix (ECM) plays many critical roles including supplying information and signals to the surrounding cells and providing structural support [1]. There are some molecules that are secreted by cells into the ECM to control different biological activities at the tissue or cellular level. Matrix metalloproteinases (MMPs) are among the key molecules that regulate different molecular and biological events in the ECM. MMPs are a family of zinc-dependent proteolytic enzymes involved in the degradation of ECM components. MMPs were firstly named based on their substrates, until it became clear that each MMP has multiple substrates. Therefore, MMPs were classified into different groups based on their domain structure, and the nomenclature was changed to a numerical system [2]. MMPs are classified into several subgroups based on their substrate preference or domain structure. Collagenases (MMP-1, MMP-8, MMP-13, and MMP-18) cleave fibrillar collagen types I, II, and III, and they can cleave other ECM proteins. Gelatinases (MMP-2 and MMP-9) have high activity against gelatin, and degrade other ECM molecules including collagens, laminin, and aggrecan. Stromelysins (MMP-3, MMP-10, and MMP-11) digest a number of noncollagen ECM molecules, and their domain arrangement is similar to that of collagenases. The membrane-type MMPs (MT-MMPs) (MMP-14, MMP-15, MMP-16, MMP-17, MMP-24, and MMP-25) are intracellularly activated transmembrane molecules, and their active forms are expressed on the cell surface. There are other less characterized members including MMP-7, MMP-12, MMP-19, MMP-20, MMP-22, and MMP-23 [3]. Most of the MMPs contain the single peptide, prodomain, catalytic domain, hinge domain, and hemopexin domain. The MT-MMPs have an extra domain called the transmembrane domain or GPI-anchored domain, which are integrated in the plasma membrane [2].

MMPs are important in a wide variety of developmental processes including mediation of cell–cell adhesion, tissue remodeling, cell migration, invasion, proliferation, and apoptosis [3]. MMPs can cleave growth factor binding proteins or latent growth factors which may regulate their synthesis and release from inside the cell [4, 5]. They are regulated by several MMP-specific inhibitors called tissue-specific inhibitors of metalloproteinases (TIMPs) [4, 5]. MMPs and TIMPs have a very critical role in matrix remodeling that takes place during the regeneration of any tissue [4, 5]. The activity and function of MMPs suggest their involvement in different cellular activities during cell development. This fact opens the door to investigate the involvement of these enzymes in the migration, proliferation, and differentiation of mesenchymal stem cells (MSCs).

Regenerative medicine is a promising approach that involves stem cells and microenvironmental factors to stimulate differentiation into different lineages. MSCs have great potential as a source of cells for cell-based therapy because of their ability for self-renewal and differentiation into functional cells. MSCs are adult stem cells of stromal origin that can be found in different biological sources not only in bone morrow, but also in other various tissue sources [6]. They are multipotent cells and are known to migrate from bone morrow to different tissues with different compositions of ECM, which justify the studies to examine their susceptibility to matrix variation [7]. Moreover, it has been demonstrated that MMPs have critical role in the differentiation of MSCs to adipocytes, osteocytes, and chondrocytes [4]. MSCs also interact with exogenous MMPs at their surface and activate proMMP-2 and proMMP-13, regulating the pericellular localization of MMP activities [6]. They have the capability to regulate exogenous MMP-2 and MMP-9 by the expression of TIMP-2 and TIMP-1, protecting the perivascular niche from their high levels [8].

There is increased interest in the use of MSCs as therapeutic tools to treat different diseases because of their ease of isolation, immune capability, expansion, and proliferative and differentiation potency [9]. Recently, the focus of many studies on MSCs is the connection between cells and matrix signals. However, the mechanisms that regulate the proliferation, migration, self-renewal, and differentiation of MSCs are still not fully understood [10]. Identifying the molecules that regulate the fate of MSCs is an important approach to understand how these cells can be controlled and thus used as therapeutic tools. In this study, we critically reviewed the findings on the effect of MMPs in angiogenesis, migration, and proliferation of MSCs. We also reviewed the role of MMPs on the multilineage property of multisource-derived MSCs to differentiate into adipocytes, chondrocytes, and osteocytes.

## Role of MMPs in the differentiation of MSCs

### Adipogenic differentiation

MMPs have been implicated in regulating adipogenic differentiation of MSCs. MMPs and TIMPs have a crucial role, either negative or positive, in the differentiation of adipoblasts, which are derived from MSCs. Formation of adipose tissue involves triggering of MSCs to differentiate toward a preadipocyte lineage and finally to an adipocyte lineage. This differentiation involves an increase in the production and secretion of some MMPs [10]. It was hypothesized that the formation of basement membrane and remodeling of pericellular basement membrane are required for the differentiation of MSCs to adipocytes. Sillat and his group [11] studied the expression of basement membrane collagen type IV during the adipogenic differentiation of MSCs. These investigators found an increase in the expression of collagen type IV by MSCs during adipogenesis, and the expression of MMP-9 and TIMP-2 was significantly induced during the differentiation. MMP-2 expression was localized in the cytoplasm and close to the nucleus in the differentiated and undifferentiated MSCs [11]. There was no significant upregulation of MT1-MMP expression. These data suggest that MSCs express collagen type IV, and secrete type IV collagenase (MMP-9) to remodel it during adipogenic differentiation [11]. Although TIMPs have been known to inhibit MMPs, the interaction of proMMP-2 with TIMP-2 is required for the activation of proMMP-2. TIMP-2 binds the catalytic domain of MT1-MMP on the cell surface, and the C terminal of TIMP-2 recruits the endogenous proMMP-2 to the cell surface and binds to it, forming the proMMP-2/TIMP-2/MT1-MMP complex. Once this complex is formed, an active MT1-MMP closely located to the complex cleaves the prodomain and activates proMMP-2 [2]. However, mouse embryonic cells overexpress MMP-2, and downregulate the expression of MMP-11 during adipogenic differentiation in adipogenic differentiation medium. Additionally, MMP-11-defecient mouse embryonic fibroblasts showed increased expression of adipocyte markers compared with the wild type, indicating the inhibitory effect of MMP-11 on the adipogenic differentiation [12]. In a recent study, murine embryonic fibroblasts derived from MMP-2-deficient mice showed impaired adipogenic differentiation as compared with control murine embryonic fibroblasts derived from wild-type mice. The MMP-2-deficient murine embryonic fibroblasts showed significant decreases in intracellular lipid content and expression of adipogenic markers [13]. Moreover, the study also showed that selective knockdown of MMP-2 in preadipocytes resulted in impaired adipogenic differentiation, while the overexpression of MMP-2 resulted in significantly enhanced adipogenic differentiation [13]. A similar study investigated the role of MMP-9 in adipogenic differentiation of preadipocytes. The results showed that the silencing of MMP-9 did not affect the intracellular lipid content or expression of adipocyte specific markers, indicating that MMP-9 has no effect on the differentiation of preadipocytes into adipocytes [14]. However, several studies that examined the role of MMP-9 in the adipogenic differentiation suggested the positive regulatory role of MMP-9 in this process. It is worth mentioning that studies with MMP inhibitors lack specificity to a single MMP, and can inhibit MMP-2, MMP-9, and other proteases. In contrast, this study was performed using MMP-9 shRNA silencing, which did not show significant inhibitory effect on the expression or activity level of MMP-2. Moreover, broad-spectrum inhibitors can also inhibit a disintegrin and metalloproteinases (ADAMs), affecting different physiological processes. The expression of MMP-9 in this study was upregulated in the first 2 days of stimulation for adipogenic differentiation, followed by a rapid decrease until the end of differentiation, suggesting that the upregulation of MMP-9 does not necessarily have a key role in this process [14]. In another study, treating preadipocytes with a selective inhibitor for MMP-13 or silencing MMP-13 by siRNA resulted in reduced adipogenic differentiation, suggesting the important role of MMP-13 in adipogenesis [15].

Several MMPs and TIMPs have been shown to have a positive or negative regulatory effect on the adipogenic differentiation, indicating their distinct roles during the commitment of differentiated cells. MMP-2 and MMP-9 were found to be upregulated during the differentiation of preadipocytes to adipocytes, and the inhibition of MMPs resulted in the suppression of adipogenic differentiation [16]. Another study reported that MMP-13 was significantly upregulated during adipogenic differentiation, while there was a significant downregulation in the expression of TIMP-1, TIMP-2, and TIMP-3. The findings from this study suggested an adjustment in the expression of MMPs and TIMPs in the early stage of differentiation to trigger the process toward a specific lineage [17]. It has been also reported that MMP-9 regulates the adipogenic differentiation by controlling the expression of adipogenic cytokines, including IL-1β, IL-8, and IL-6 [18]. Furthermore, the expression of MMP-2 and MMP-9 was upregulated during the adipogenic differentiation of preadipocytes with a decrease in the expression of TIMP-1 [19]. The effect of high-temperature requirement proteases A1 (HTRA1) was found to have a regulatory effect on MSCs undergoing adipogenic differentiation through its impact on the expression of MMPs. The treatment of MSCs with HTRA1 during adipogenic differentiation showed a significant increase in mRNA expression of MMP-1, MMP-2, MMP-3, MMP-9, and MMP-13. At the protein level, the expression of MMP-3 and MMP-13 was significantly increased during adipogenic differentiation of MSCs with HTRA1. Moreover, MMP inhibitors (NNGH and CL-82198) for MMP-3 and MMP-13 during adipogenic differentiation increased the level of collagen type IV in HTRA1-treated MSCs, suggesting the important role of HTRA1 in inducing the expression of MMP-3 and MMP-13, and in adipogenic differentiation of MSCs [20]. Fibronectin was found, in vitro, to be produced first among all ECM components during adipogenesis. Different types of collagen were secreted by differentiated cells and degraded during different stages of the adipogenic differentiation. This suggests that there are different MMPs and TIMPs which are expressed during the adipogenic differentiation to participate in the progress of this process by either remodeling and/or degrading the ECM components [21]. The involvement of MMPs and TIMPs, which have so far been studied in the regulation of adipogenic differentiation, reveals a crucial role of these enzymes in the differentiation of MSCs to adipocytes.

### Chondrogenic differentiation

ECM remodeling is a crucial process during skeletal formation, which is required for chondrocyte progenitor cells to undergo differentiation [10]. Although the role of MMPs in vivo during chondrogenic differentiation is still not clear, in-vitro studies have shown their crucial role during this process. Chondrogenic differentiation of human MSCs increases with the increase in collagen concentration, yielding increased matrix production [22]. This may indicate a direct or indirect role of MMPs during the differentiation process, since they are important candidate molecules for communicating signals and promoting the turnover of ECM molecules.

**Fig. 1:**
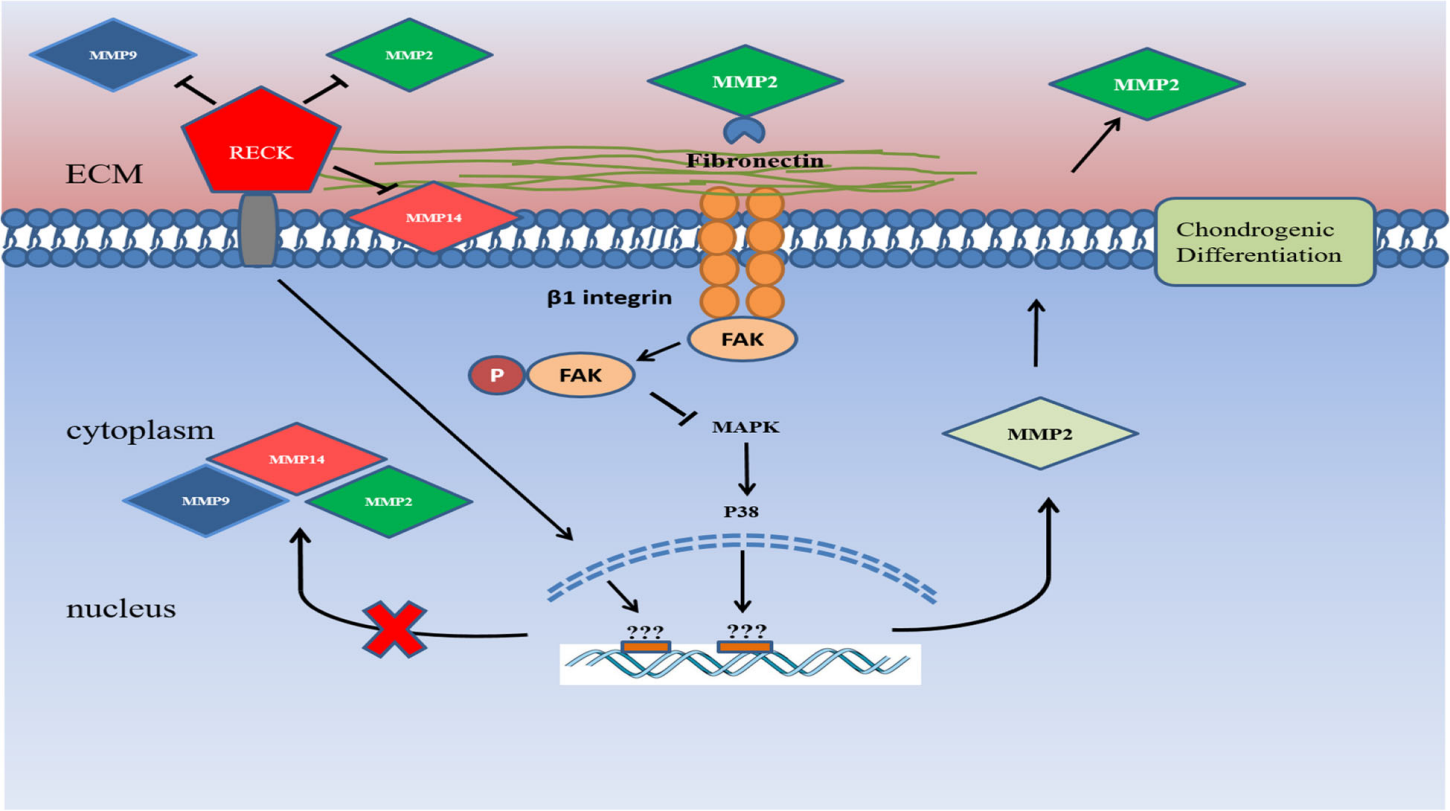
MMPs and chondrogenic differentiation of MSCs. The upregulation of reversion-inducing-cysteine-rich protein with Kazal motifs (*RECK*) during the late stages of chondrogenic differentiation downregulates the expression of MT1-MMP (MMP-14), MMP-2, and MMP-9, and directly inhibits them through its enzymatic activity. Expression of MMP-2 is upregulated by the increase in P38 activity resulting in stimulation of the chondrogenic differentiation of MSCs. MMP-2 also regulates the association of FAK and β1 integrin through its degradation of fibronectin. This degradation results in the inhibition of MAPK pathway through P38 to stop the production of more MMP-2 at late stages of the process. *ECM* extracellular matrix, *MMP* matrix metalloproteinase, *FAK* focal adhesion kinase

Jin and colleagues [23] investigated the role of MMP-2 in chondrogenic differentiation of MSCs. Their data showed that the silencing of MMP-2 by siRNA impaired chondrogenic differentiation, and increased the protein level of fibronectin and β1 integrin. Treatment with a MMP-2 activator resulted in the activation of chondrogenesis. These results also indicated that the downregulation of P38 mitogen-activated protein kinase (MAPK) resulted in the inhibition of MMP-2 expression [23]. Moreover, MMP-2 is involved in the chondrogenic differentiation of MSCs via downregulation of focal adhesion kinase (FAK)–β1 integrin interaction, which leads to phosphorylation of FAK (Fig. 1) [23]. In another study, a group of scientists investigated the role of the degradation of MMPs on the chondrogenesis of MSCs. The study investigated the chondrogenic differentiation in MMP-sensitive hydrolytically hydrogel scaffold in comparison with MMP-insensitive hydrogels [24]. They found that the MSCs in the MMP-sensitive hydrogels had more morphological changes and chondrogenic markers compared with MSCs in the MMP-insensitive hydrogel group which remained round in shape [24]. A similar study was carried out using degradable or nondegradable polyethylene glycol gels. The human MSCs were evaluated for their proliferation, morphology, and chondrogenic differentiation in the two different gels. MSCs in the degradable gel showed more morphological changes and chondrogenic differentiation compared with MSCs in nondegradable gel after 21 days [25]. These studies revealed that the degradation of MMPs enhances the chondrogenic differentiation of MSCs by allowing the morphological changes and increasing the contents of glycosaminoglycans (GAG) and expression of chondrogenic markers. A chondrogenic cell line (ATDC5) was examined for chondrogenic differentiation, and the expression of MMP-2, MMP-9, and MT1-MMP was upregulated during the early stages of chondrogenic differentiation with a downregulation in the expression of reversion-inducing cysteine-rich protein with Kazal motifs (RECK) [26]. However, RECK was shown to be upregulated in the later phase of differentiation. The knockdown of RECK revealed a suppression in the later ECM accumulation in the cartilaginous nodules. The data suggested that RECK and MMPs have an important effect in the chondrogenic differentiation through regulating tissue morphogenesis (Fig. 1) [26]. Previous studies showed that the upregulation of MMP-13 occurs during chondrogenic differentiation, and MMP-13 has been found to be upregulated in patients with osteoarthritis, suggesting a pathological role of MMP-13 in the disease process. Salinas and her group monitored the expression of MMP-13 during chondrogenic differentiation of MSCs, and they found an increased expression of MMP-13 followed by an increase in GAG content by day 9 until day 14 in vitro. The results indicate that the increase in production of MMP-13 drives the MSC differentiation to chondrocytes by degrading the cleavable components of the ECM, and regulating integrin-binding peptides [27].

Although there is no direct connection between secreted proteins and MMP activity, it is clear that MMPs play an important regulatory role during differentiation of MSCs into chondrocytes. The role of MMP-13 during skeletal repair and development is very crucial. The absence of MMP-13 affects the removal of hypertrophic cartilage during nonstabilized fracture healing by MSCs. MMP-13 produced by chondrocytes seems to be important in the initiation of cartilage degradation and recruitment of blood vessels [28]. MSCs can also trigger cartilage matrix remodeling by upregulating the expression of certain MMPs. MMP-13 was found to have an important role in the later stages of chondrogenic differentiation of MSCs by degrading the main components of the cartilaginous matrix, aggrecan and type II collagen [29]. Another study investigated the effect of ascorbic acid 2 phosphate and type 1 atelocollagen on the increase in collagen type II accumulation in scaffold-free cartilage-like cell sheets prepared using human bone marrow-derived MSCs. The study indicated that the synthesis of collagen type II increased after ascorbic acid 2 phosphate and type 1 atelocollagen supplementation to chondrogenic differentiation media [30]. Moreover, the gene expression of MMP-13 was reduced compared with control, suggesting a role for MMP-13 in the degradation of collagen type II during chondrogenic differentiation [30]. Stimulation of MSCs in chondrogenic media with IL-β1 resulted in increased expression of MMP-2, MMP-3, and MMP-13 while IL-6 downregulated the expression of MMP-3 and MMP-13 compared with control without stimulation [31]. The knockdown of discoidin domain receptor 1 in adipose-derived MSCs (AMSCs) resulted in increased expression of chondrocyte specific markers, and impaired expression of runt-related transcription factor 2 and MMP-13 during chondrogenic differentiation [32].

### Osteogenic differentiation

**Fig. 2:**
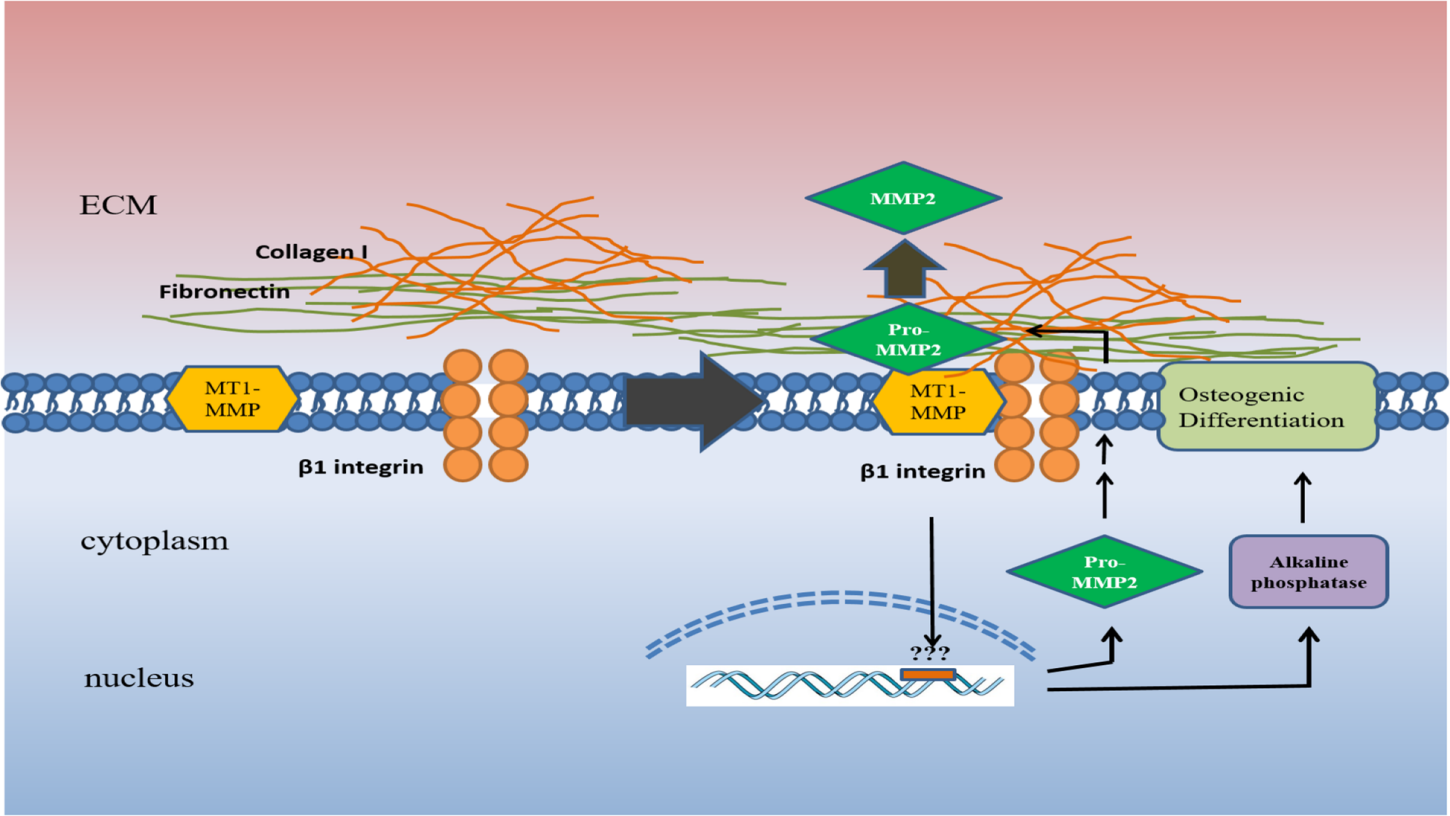
MMPs and osteogenic differentiation of MSCs. β1 integrins engage with fibronectin and collagen type I in the ECM, resulting in the formation of MT1-MMP and β1 integrin complex. This complex initiates the expression of proMMP-2 and ALP, which is required for the formation of nodules and their mineralization, and allows the activation of proMMP-2 through MT1-MMP during the osteogenic differentiation. *ECM* extracellular matrix, *MMP* matrix metalloproteinase, *MT-MMP* membrane type-matrix metalloproteinase

Osteogenic differentiation and bone regeneration are characterized by ECM remodeling and hormonal and growth factor interactions. MSCs are very important cells for bone regeneration because of their capability to migrate into sites of injury and differentiate into osteocytes. Bone undergoes different physiological processes, including bone modeling and remodeling. Bone modeling leads to a change in bone shape in response to biomechanical forces. However, bone remodeling maintains bone strength through the resorption of old bones and the formation of new bones by dependent actions of osteoclasts and osteoblasts [33]. The differentiation of osteoblasts and bone formation are controlled by bone morphogenetic protein (BMP) and wingless (Wnt) signaling pathways, leading to the synthesis of collagen type I, whereas osteoclast differentiation is controlled by the receptor activator of nuclear factor kB (RANK) signaling pathway through macrophage colony-stimulating factor (MCSF) [34]. The role of MMPs on osteogenic differentiation has been studied, and their effect in osteoclastic resorption has been supported by studies indicating that MMP inhibition may block the osteoclastogenesis. Specific inhibition of several MMPs showed an important role for MMP-13 in MSC differentiation to osteocytes. The inhibition of MMPs by a broad-spectrum inhibitor revealed a huge alteration in osteogenic differentiation of MSCs [35]. These findings suggest that MSC differentiation is correlated with MMP and TIMP activity and the balance between the two molecules [35]. In another study, a broad-spectrum inhibitor for MMPs was used to determine the need for ECM remodeling during osteogenic differentiation of preosteoblasts, and the data revealed that MMP activity is necessary for the transition to osteoblasts [36]. The absence of MMP-13 affects the remodeling process during osteogenesis and after bone marrow transplantation, suggesting its key role in the osteogenic differentiation. This indicates that MMP-13 is involved in skeletal repair, and acts in the early stages of ECM degradation prior to invasion of osteoclasts and blood vessels [28]. Manduca and colleagues [37] have investigated the role of MMPs in osteogenic differentiation. In this study, the plating of preosteoblasts on MMP-inducing substrata resulted in the formation of β1 integrin complexed with MT1-MMP. The MT1-MMP senses the surrounding microenvironment through the binding of β1 integrin and the ECM. Moreover, overexpressing MT1-MMP during osteogenic differentiation was found to upregulate alkaline phosphatase (ALP) which is involved in crystal deposition on the scaffold of collagen type I fibers in the ECM of osteoblasts. The data suggest that MT1-MMP expression is crucial in the formation of nodules and mineral deposition during osteogenic differentiation. The expression of MT1-MMP during osteogenesis allows ECM remodeling, and initiates the expression of ALP and proMMP-2 (Fig. 2) [37].

MSCs have the ability to adjust the expression phenotype of MMPs and TIMPs to modify and maintain the ECM in a structural state optimal for differentiation into a specific lineage. When human MSCs were stimulated for osteogenic differentiation, the expression of MMPs and TIMPs was adjusted to promote the structural conformation and ECM remodeling optimal to osteogenic differentiation. There was a decrease in the expression of MMP-1 and MMP-8 with an increase in the expression of TIMP-2 and TIMP-4. The results indicate that TIMPs and MMPs are upregulated or downregulated in differentiation-specific manners [38]. MSCs, in culture, express some MMPs including MMP-1, MMP-2, MMP-13, MT1-MMP, and MT3-MMP. However, MMP expression can be modulated by the composition of culture. During stimulation of MSCs for osteogenic differentiation, silencing each of the MMPs mentioned earlier showed that MT1-MMP is the only MMP that is responsible for MSC-mediated type I collagenolysis. In 3D culture, MSCs express ALP activity with inhibition of TIMP-2 activity. Silencing MT1-MMP resulted in impaired morphological change of MSCs and ALP expression, and the cells failed to undergo osteogenic differentiation. The inability of MSCs to undergo osteogenic differentiation was rescued by adding mouse MT1-MMP [39]. In 2D culture, MT1-MMP silencing did not affect the osteogenic differentiation of MSCs [39]. To study the role of extracellular signal-related kinase (ERK) in collagen-induced osteogenic differentiation of MSCs, a mitogen-activated protein kinase (MEK) inhibitor was used in 2D and 3D cultures. The data indicated that osteogenic differentiation is ERK-mediated differentiation [40]. Moreover, the osteoblastic differentiation of MSCs was increased after X-ray irradiation with a decrease in the expression of collagen I and II, which was due to significant increase in the expression of MMP-3 and MMP-13 [41]. However, another study examining the expression profile of mice bone marrow-derived MSCs showed that the expression of MMP-13, MMP-3, and MMP-2 was downregulated during osteogenic differentiation [42]. To study the role of sox9 on the osteogenic differentiation of AMSCs, sox9 was knocked down by shRNA, which resulted in increased expression of osteocalcin, vascular endothelial growth factor alpha (VEGF-α), and MMP-13 in the early stages of differentiation [43].

### Endothelial differentiation

MSC therapy can be used for regeneration of the injured endothelial layer. The endothelial differentiation of MSCs derived from different sources has been reported [44–50]. Even though MMPs were initially reported to have an ability to degrade the ECM, MMPs are now known to regulate many biological processes, being involved not only in physiological events but also in pathological processes [51]. MMPs and their inhibitors have important roles in mediating cell–cell adhesion, cell migration and invasion, cell proliferation, apoptosis, and tissue remodeling [1]. MMPs promote the release of ECM-bound or cell surface-bound cytokines, which then regulate the differentiation of stem cells [52, 53]. It has been suggested that ECM signaling regulates endothelial cell (EC) morphogenesis and induction of angiogenesis. ECM signaling can therefore be coincident with degradation of the ECM and exposure of ECs to collagen type I [1]. The ECM remodeling by MMPs has a key role in angiogenesis, migration, and morphogenic differentiation of ECs [54]. Moreover, it was demonstrated that the recruitment of stem and endothelial progenitor cells from the quiescent niche in bone marrow is dependent on MMP-9, which enhances cell mobility and rapid differentiation [55]. Therefore, MMP expression could be transiently manipulated to induce differentiation of MSCs into ECs. The role of MMPs in the differentiation of MSCs to different lineages has been reported previously. To date, there are no published data that show the relationship between MMPs and endothelial differentiation of MSCs. In our laboratory, we studied the expression of a group of MMPs and TIMPs to evaluate their role on the differentiation of AMSCs to ECs. The study is still under investigation and is designed to evaluate the expression and effect of MMPs and TIMPs at three time points during EC differentiation of AMSCs.

## MSCs express MMPs and TIMPs to regulate different processes

### Angiogenesis

The role of MMPs in angiogenesis is important for therapeutic angiogenesis and tissue engineering to enhance blood vessel growth and nutrient delivery after myocardial infarction. TIMP-3, for instance, was found to block VEGF receptors and inhibit VEGF-mediated angiogenesis [56]. Further, the downregulation of MMP-2 and MT1-MMP expression by UVA irradiation was found to inhibit angiogenesis [57].

MSCs transfected with VEGF/HGF delivered through the coronary vein into infarcted pig heart were shown to increase cardiac function by improving the angiogenesis process in the injured tissue [58]. At present, MSCs are known for their ability to influence the behavior of ECs, and promote their formation of capillary-like structures. MMPs that are expressed by MSCs can contribute to that role during the interaction of MSCs and ECs [4]. Glaeser and colleagues [59] demonstrated that the inhibition of MMP-2 attenuated the tube-like formation. Under mechanical stimulation by MSCs, the secretion of proMMP-2 and the activity of MMP-2 were induced and, as a result, this induction contributes to angiogenesis stimulation. It was also demonstrated in another study that mechanically stimulated MSCs have higher angiogenic properties with an observed enhancement in the activity of MMP-2 compared with unstimulated MSCs [4, 33].

MSCs have angiogenic properties that involve degradation of ECM. This was supported by an in-vitro study that showed an induction of MSC capillary-like structure formation in hypoxic culture condition. Further, it was noticed that MT1-MMP expression was significantly upregulated, and the inhibition of MT1-MMP reduced the capability of MSCs to form capillary-like structures. These data suggest that MT1-MMP has an important regulatory role in ECM remodeling and MSC capillary-like structure formation [60]. The addition of MSCs to ECs in 3D fibrin matrices resulted in an increased matrix density and network formation. It was also predicted that MMP-2, MMP-9, and MT1-MMP expressions were upregulated after the addition of MSCs. However, the data revealed that only MT1-MMP among these proteases has a crucial role in facilitating the angiogenesis process. These results suggest that MT1-MMP can act locally to mediate ECM degradation and allow the release of matrix-bound growth factors that are important for angiogenesis [61]. Intravenous administration of bone marrow-derived MSCs after middle cerebral artery occlusion in a rat model resulted in increased activity of MMP-2 and vascular density, and in reduced infarction volume suggesting the important role of MMP-2 activity and MSCs in promoting angiogenesis [62]. Moreover, coculturing of human amniotic MSCs or bone marrow-derived MSCs with human umbilical vein ECs, seeded in fibrin gels and injected subcutaneously into mice, was done to evaluate their angiogenic capacity. The results showed increased expression of platelet endothelial cell adhesion molecule (PECAM) and vessel-like structure density compared with human umbilical vein EC monoculture [56]. The in-vitro experiment showed significantly increased formation of capillary-like structures in coculture with a significant increase in the activity of MMP-2 and MMP-9 compared with human umbilical vein EC monoculture [63]. Inhibition of MMPs by broad-spectrum inhibitor in platelet fibrin gel and cardiac stem cells, when cotransplanted into rat hearts with myocardial infarction, resulted in impaired functional and structural benefits of cell gel in treating myocardial infarction, and reduced host angiogenesis [64]. Overexpression of miR-195a-3p, which is highly expressed in MSCs, was found to decrease angiogenesis through the inhibition of MMP-2 and activation of anti-angiogenic factor pigment epithelium-derived factor, suggesting a therapeutic potential of miR-195a-3p as an inhibitor for angiogenesis [65].

### Proliferation

In order to use MSC transplantation in tissue regeneration as a therapeutic tool, we need to know the molecules and signaling pathways that participate and control MSC expansion. The remodeling of the pericellular matrix of MSCs is a prerequisite for these cells to proliferate [66]. The role of a histone deacetylase inhibitor (valproic acid) in regulating MSC proliferation was examined. The data revealed an increase in the expression level of proliferation markers associated with an increased expression of proMMP-2 and MMP-2, suggesting an important role for MMP-2 during this process [67]. Shi and his group [66] studied the role of MMPs in the proliferation of MSCs using MT3-MMP-deficient mice and found growth inhibition as a result of a decrease in MSC viability. This finding suggested that MT3-MMP has a key role in collagenolysis by allowing other surrounding cells to degrade high-density fibrillar collagen and make the ECM permissive for proliferation. In MT1-MMP and MT3-MMP double-deficient mice, the results indicated a severe effect of MT1-MMP deficiency in bone formation. Altogether, the results revealed that the deficiency of both MT1-MMP and MT3-MMP leads to severe defects as a result of lack in collagenolytic activity that is required for ECM remodeling and cell proliferation [66].

MMPs and TIMPs are important for ECM remodeling, affecting MSC behavior during their proliferation. Broad-spectrum inhibition of MMPs affects the proliferative capacity of MSCs. Protein and mRNA analysis detected the expression of MMP-2, MMP-3, MMP-10, MMP-11, MMP-13, MT1-MMP, and TIMP-2 in MSCs. Mechanical stimulation of MSCs resulted in increased protein expression, but not mRNA expression, of MMP-2, MMP-3, MMP-13, and TIMP-2. These findings suggest that secretion or activation of these MMPs is affected by posttranscriptional regulation in response to mechanical stimuli, and MMP/TIMP balance is important in the regulation of this process [33]. Broad-spectrum inhibitors for MMPs resulted in a significant decrease in the proliferative activity of MSCs [33]. Moreover, it was found that MT1-MMP expression is upregulated by PDGF-BB, which resulted in an increase in the proliferative activity of MSCs. However, the inhibition of ERK1/2 and PI3K/AKT leads to the inhibition of MT1-MMP and proliferative activity of MSCs. These data revealed an important role for MT1-MMP in the invasive and proliferative activity of MSCs which are regulated by the ERK1/2 and PI3K/AKT signaling pathways [68]. Overexpression of miR-195a-3p was also found to decrease MSC proliferation through the inhibition of MMP-2 [65]. The incubation of dermal fibroblasts with conditioned medium of umbilical cord blood-derived MSCs increased the proliferation of dermal fibroblasts with an increased ratio of MMP/TIMP [69]. Microvesicles are cell-derived vesicles released by different type of cells into the ECM and fuse with neighboring cells to transfer protein and RNA. They are classified into exosomes and ectosomes [70–72]. The interactions between cancer cells, ECs, and MSCs via microvesicles were examined. MSCs were treated with microvesicles isolated from conditioned medium of ECs or cancer cells, and such treatment of microvesicles increased the proliferation and migration of MSCs together with an increase in MMP-1 and MMP-3 [73].

### Migration

In order for cells to migrate, they undergo molecular and structural changes to move from an adhesive phenotype to a migratory phenotype [3]. MMPs have an essential role in regulating the migratory activity of transformed cells. The degradation products that result from MMP proteolysis of ECM also have a mediatory effect on cell migration [2]. Migratory behavior of MSCs involves MMP activity. MSC migration was found to be impaired by a broad-spectrum MMP inhibitor [4]. MMP-2 inhibition in tumor cells was also found to decrease MSC migration through the inhibition of SDF1/CXCR4 signaling, which indicates a mediatory effect for MMP-2 in the migration of MSCs [74]. MSCs express CXCR4, MMP-2, and MT1-MMP to increase mobility and promote migration and recruitment of expanded MSCs to injured tissues [75]. Valproic acid, a histone deacetylase inhibitor, enhances MSC migration through the upregulation of both CXCR4 and MMP-2 expression [67]. Further, enhanced gene expression of CXCR4, MMP-2, and MMP-9 by heat shock protein 90 (Hsp90) leads to an increase in the migratory behavior of MSCs [76]. MMP-2 was also found to have a positive role in the transendothelial migration of MSCs, while the increase in culture confluency of MSCs along with MMP-2 siRNA inhibition decreased the transendothelial migration of MSCs and increased the expression of TIMP-3 [77]. The expression of MMP-2 can be downregulated by norepinephrine via β3-adrenergic receptor (adrb3) in MSCs and inhibits the migratory behavior of MSCs from perivascular regions to bone-forming units. Silencing adrb3 decreases the inhibition of MSC migration by upregulating MMP-2 expression and downregulating TIMP-3 expression [78]. The overexpression of miR-195a-3p was also found to impair MSC migration via the inhibition of MMP-2 [65].

Ries et al. [51] analyzed the expression of MMPs and TIMPs in bone marrow-derived MSCs. The results showed that bone marrow-derived MSCs highly express MMP-2, MT1-MMP, TIMP-1, and TIMP-2. However, silencing MMP-2, MT1-MMP, or TIMP-2 decreased MSC migration, whereas TIMP-1 knockdown promoted the process. It was also indicated that TGF-β1, TNF-α, and IL-1β upregulate the expression of MMP-2, MT1-MMP, and TIMP-2, which also lead to the activation of MSC migration. These findings revealed that MMP-2 and MT1-MMP have an essential role in MSC migration, and they can be induced by some cytokines in vivo to increase the recruitment and migration of MSCs to the injured tissue [51]. The findings of this study support the role of MMP-2 in MSC migration. In another study, a group of scientists demonstrated the effect of hypoxic culture in stimulating the migration of MSCs in vitro. They found that treating the cells with cytokine-enriched medium and hypoxic conditions induce the migratory behavior of MSCs with a downregulation in MMP-2 and upregulation in MT1-MMP expressions. The inhibition of MT1-MMP also revealed its important role in stimulating the migration of MSCs [60, 79]. Lu et al. [39] also indicated that MT1-MMP has a critical role in MSC migration by degrading and penetrating collagen type I networks. Specific inhibition of MT1-MMP impaired the migration of MSCs, and this was reversed after transfecting the cells with MT1-MMP expression vector [39]. Treatment of human umbilical cord blood-derived MSCs with arachidonic acid, which is released during injuries, was reported to induce fibronectin degradation by MT3-MMP through the phosphorylation of p38 MAPK and transcription factor Sp1, and stimulated the MSC motility. The arachidonic acid treatment also enhanced skin wound healing in vivo through induction of MSC motility by MT3-MMP degradation activity [80]. Furthermore, the incubation of dermal fibroblasts with a condition medium from umbilical cord-derived MSCs was found to enhance the migration of dermal fibroblasts with increased ratio of MMP/TIMP [69]. Several studies have reported the MSC migration to the sites of tumor, suggesting the role of specific signals in activating MSC migration. The incubation of MSCs with conditioned medium of hepatoma cell culture was found to increase MSC migration by significantly increased levels of MIP-1δ, MIP-3α, and MMP-1, suggesting the dependency of MSC migration on MMP-1 [81]. In a recent study, the effect of a conditioned medium of bone marrow-derived MSCs on the proliferation and photo-aging of dermal fibroblasts after UV exposure was investigated. The results showed that MSC conditioned medium significantly reduced the UV-induced expression of MMP-1 and increased procollagen synthesis, suggesting a therapeutic potential of MSC-conditioned medium in preventing dermal damage through MMP-1 downregulation [82].

A comparison between the expressions of some genes in the MSC isolates showed that the increase in MMP-1 expression was associated with highly migrating MSCs compared with those with poor migration [83]. The inhibition of MMP-1 revealed a significant decrease in MSC migration. Similarly, blocking the interaction of MMP-1 and protease-activated receptors (PAR1) resulted in the inhibition of MSC migration [83]. Inhibition of hypoxia-inducible factor 1 alpha (HIF-α) in MSCs was shown to decrease the expression of MMP-1 and MMP-3, resulting in poor migration. These findings suggest the important role of MMP-1 in addition to MMP-3 in MSC migration via HIF-α [84].

## Conclusion

MSCs are potent cells for normal regenerative processes because of their ability for self-renewal and differentiation into different lineages [79, 80]. Commitment of MSCs to differentiate into a specific lineage, proliferate, or migrate is regulated by many factors in the local tissue microenvironment. Each of these types of cells is associated with expression of a distinct set of proteins. However, there are many challenges to dissect the underlying regulatory mechanisms in the differentiation and function of MSCs. These challenges include the identification of MMPs and TIMPs as well as the crosstalk between signaling pathways that promote self-renewal, migration, proliferation, and lineage differentiation in MSCs. The ability of MMPs to regulate and/or change the function and activity of different proteins and receptors suggest that they may also be involved in controlling the commitment of MSCs. Since MMPs degrade all of the components of ECM, their role in controlling and regulating matrix functions has been studied extensively. The potential of MSCs to differentiate into a particular mesenchymal lineage relies upon upregulation or suppression of genes specific to this lineage.

**Fig. 3:**
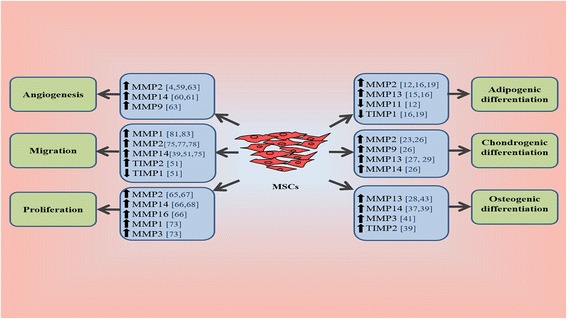
MMP participation in the fate of MSCs. The proliferation, migration, angiogenesis, and differentiation of MSCs are promoted by an increase or decrease in the expression of specific MMPs and/or TIMPs. *MMP* matrix metalloproteinase, *MSC* mesenchymal stem cell, *TIMP* tissue-specific inhibitor of metalloproteinase

In summary, the differentiation of MSCs into each of these cell lineages is promoted by specific MMPs and/or TIMPs associated with a specific cell lineage. MMP-9, MMP-2, MMP-11, MMP-13, and TIMP-1 are key regulators that have a crucial role in the differentiation of MSCs toward adipocytes. MMP-13 and MT-1MMP, in addition to MMP-2 and MMP-9, play a key role in the differentiation of MSCs to chondrocytes. MMP-1, MMP-3, MMP-13, and MT1-MMP are the MMP regulators for osteogenic differentiation of MSCs (Fig. 3). Moreover, TIMP-2 was also found to have a role in this differentiation. Both MMP-2 and MT1-MMP have a key role in the angiogenesis, proliferation, and migration of MSCs. In addition, MMP-1, MMP-3, and MT3-MMP have a regulatory effect in MSC proliferation, while MMP-9 was found to have an effect on the angiogenic activity of MSCs. MMP-1, MMP-3, TIMP-1, and TIMP-2 also play an important role in MSC migration (Fig. 3).

There are only a few studies addressing the mechanisms behind the effect of MMPs on MSC differentiation. The binding associations between MMPs and cell surface receptors suggest a direct effect of MMPs to trigger the signaling pathways involved in MSC migration, proliferation, growth, and differentiation. MMP-2 can bind to αVβ3 on the cell surface of MSCs, and the mesenchymal invasive behavior may depend on this binding. In epithelial cells, the binding of proMMP-2 to αVβ3 resulted in increased expression of VEGF and angiogenesis through the PI3K/AKT pathway [85], suggesting a potential mechanism for the effect of MMPs on the angiogenic activity of MSCs. Moreover, the binding of proMMP-9 to α4β1 integrin and CD44 was found to induce chronic B-lymphocytic leukemia survival through the activation of an intracellular signaling pathway, which results in STAT3 phosphorylation and induced myeloid leukemia cell differentiation protein (Mcl-1) upregulation [85]. It has also been reported that angiotensin II (Ang II) induces the expression of MMP-2 and MMP-14 through the activation of the MAPK signaling pathway [86], indicating a critical role of Ang II in the expression of MMP-2 and MMP-14. The binding of TIMP-1 to CD63 on the plasma membrane of MSCs promotes the degradation of β-catenin, and knockdown of TIMP-1 stimulates the activity of β-catenin and promotes osteogenic differentiation through the Wnt/β-catenin signaling pathway [87]. In sum, this represents possible interactions between MMPs and MSC surface molecules or/and intracellular signaling pathways involved in a major biological events in MSCs. However, there is still a lack of information about the mechanisms that regulate the expression and effective roles of MMPs in the differentiation of MSCs into different mesenchymal lineages. MMPs are critical molecules in a wide variety of developmental processes including mediating cell–cell adhesion, tissue remodeling, cell migration, invasion, proliferation, and apoptosis [3]. The activity and function of MMPs suggest their involvement in different cellular activities during cell development. They have a critical role in matrix remodeling that takes place during the regeneration of any tissue [4]. Further research is warranted in the field of MMPs and their regulatory effects on the fate of MSCs. However, the complex nature of the proteolytic environment makes it difficult to study the MSC-MMP and MSC–matrix interactions. These interactions need further examination to control the fate of MSCs and utilization of such knowledge for translational purposes. Overall, the findings support the crucial role of MMPs in promoting the differentiation, angiogenesis, proliferation, and migration of MSCs. However, the conflicting data warrant more careful and controlled studies for better understanding of the process.

## Abbreviations

AMSC: Adipose-derived mesenchymal stem cell
ALP: Alkaline phosphatase
EC: Endothelial cell
ECM: Extracellular matrix
ERK: Extracellular signal-related kinase
FAK: Focal adhesion kinase
HIF-α: Hypoxia-inducible factor 1 alpha
Hsp90: Heat shock protein 90
GAG: Glycosaminoglycan
HTRA1: High-temperature requirement proteases A1
MSC: Mesenchymal stem cell
MMP: Matrix metalloproteinase
MT-MMP: Membrane-type matrix metalloproteinase
PECAM: Platelet endothelial cell adhesion molecule
PAR1: Protease-activated receptor1
RECK: Reversion-inducing cysteine-rich protein with Kazal motifs
TIMP: Tissue-specific inhibitor of metalloproteinase
VEGF: Vascular endothelial growth factor
Adrb3: β3-adrenergic receptor
